# Acute renal morbidities with obstetrical emergencies: An important women health issue

**DOI:** 10.12669/pjms.333.12233

**Published:** 2017

**Authors:** Meharun-Nissa Khaskheli, Shahla Baloch, Aneela Sheeba, Sarmad Baloch, Fahad Khan, Mohammad Rafique Ansari

**Affiliations:** 1 Dr. Meharun-Nissa Khaskheli, MBBS, FCPS. Associate Professor, Department of Obstetrics & Gynaecology, Liquat Univeristy of Medical & Health Sciences Jamshoro, Sindh, Pakistan; 2 Dr. Shahla Baloch, MBBS, DGO, FCPS. Associate Professor, Department of Obstetrics & Gynaecology, Liquat Univeristy of Medical & Health Sciences Jamshoro, Sindh, Pakistan; 3 Dr. Aneela Sheeba, MBBS, DMRD, FCPS. Assistant Professor, Department of Radiology, Liquat Univeristy of Medical & Health Sciences Jamshoro, Sindh, Pakistan; 4 Dr. Sarmad Baloch, MBBS. House Officer, Medical Department, Liquat Univeristy of Medical & Health Sciences Jamshoro, Sindh, Pakistan; 5 Dr. Fahad Khan, MBBS. House Officer, Medical Department, Liquat Univeristy of Medical & Health Sciences Jamshoro, Sindh, Pakistan; 6 Dr. Mohammad Rafique Ansari, MBBS, FCPS. Assistant Professor, Department of Nephrology, Liquat Univeristy of Medical & Health Sciences Jamshoro, Sindh, Pakistan

**Keywords:** Acute renal morbidities, Mortality, Obstetrical emergencies, Types, Severity

## Abstract

**Objective:**

To observe the impact of acute renal morbidities with obstetrical emergencies on maternal health.

**Methods:**

In this study pregnant women between 28-40 weeks gestational period and delivered women in their puerperal period up to 42 days after delivery having acute renal problems associated with obstetrical emergencies were included. Pregnant and delivered women with obstetrical emergencies and associated other morbidities were excluded. These women were registered on the predesigned proforma after taking written informed consent and taking approval from institutional ethic research committee. The data was collected and analyzed on SPSS version 21.

**Result:**

Out of these 196 total registered women, majority of these women 81(41.32%) were between 21-30 years of age and multiparous women with parity four and above were 83(42.34%). Commonest presenting symptoms were generalized oedema 123(62.75%) and oligouria 92(46.93%). Frequent obstetrical emergencies observed were pre-eclampsia 53(27.04%), post partum haemorrhage 48(24.48%) and ante partum haemorrhage 36(18.36%) women. The complete recovery was observed in 86(43.87%) women, while mortality was seen in 56(28.57%) women.

**Conclusion:**

Renal morbidities were more frequently observed in obstetrical emergencies leading to high morbidity and mortality rate.

## INTRODUCTION

Acute renal failure is a clinical syndrome characterized by an abrupt decrease in the glomular filtration rate, rising in plasma urea and creatinine levels, and urine out put less than 300 ml in 24 hours. The obstetrical acute renal morbidities, a serious maternal health issue mainly concerned with the life of mother.^1^ There is a decrease in the rate of acute renal morbidities in the developed countries because of the improvement of the obstetrical care, and decrease in the infection rate.^2^ Under developed world is still facing the same situation. The incidence of obstetric related acute renal failure in developing countries like Pakistan hasnot changed significantly. There is no such local data available in the past to compare with. Only few scanty studies are available which showed pregnancy related acute renal failure (ARF) rate 7-10%.^3^ However in recent reports published from developing countries, the frequency of obstetric acute renal morbidity have been reported to be varying between 4-15%.^4^

In recent years the rates are increased in both Canada and the United States.^5^ In Canada, obstetric acute renal failure increased significantly, from 1.6 per 10 000 deliveries in 2003 to 2.3 per 10 000 deliveries in 2007, whereas the rate in the United States increased from 2.3 in 1998 to 4.5 per 10 000 deliveries in 2008.^6^ This increase is an alarming sign, because obstetric acute renal failure is associated with maternal morbidity as well as fatality rate corresponding to 2.9%.^1^ Major risk factors for obstetric acute renal failure includes chronic hypertensive disease, pre-eclampsia, postpartum haemorrhage, ante partum haemorrhage, sepsis, and other infections.^7^ The most commontype of obstetric hemorrhage is postpartum hemorrhage (PPH), mainly primary.^8^

Invasive hemodynamic monitoring and mechanical ventilation may be indicated in the event of acute hemorrhage requiring massive fluid resuscitation. Disseminated intravascular coagulopathy, acute kidney injury, and lung injury can result from hemorrhage and protracted hypovolemia.^9^ The acute obstetrical renal morbidity is a serious maternal health issue endangering the life of the women, such types of the studies will help in taking timely appropriate measures in managing obstetrical emergencies and proper handling the high risk cases as well as managing the renal morbidities with multidisciplinary approach so as to decrease the mortality rate. Our objective was to observe the impact of acute renal morbidities with obstetrical emergencies on maternal health.

## METHODS

This study was carried out at the Department of Obstetrics and Gynaecology of Liaquat University of Medical and Health Sciences Jamshoro from 1^st^ September 2013 to 31^st^ August 2016. This was an observational study on 196 pregnant and delivered women with obstetrical emergencies and renal morbidities.

The renal morbidities were diagnosed on the basis of history of sudden oliguria (urine out put <300 ml/ 24 hours), or with anuria and increase in the level of renal function tests, while the pregnant and delivered women with liver disorders, cardiac and respiratory problems were excluded. Sample size was estimated by formula (N= (Z)^2^> (pq)/e^2^. Confidence interval 95%. Sampling technique is Non –Probability Purposive. These women were registered on the predesigned proforma after taking written informed consent and approval from Institutional ethic research committee. The study variables were the age of women, gestational period, in delivered women postnatal period, symptomatology, general, obstetrical examination finding. Investigations complete blood count, renal functions tests, ultrasonographic record. All these women were managed with institutional management protocol and where ever required with multidisciplinary approach. The management out come was recoded in terms of complete recovery when renal functions were with in normal limit, partial recovery when there was improvement in renal functions but was not with in normal limit and mortality.

The data was collected and analyzed on SPSS version 21 (IBM SPSS 21, INC USA, 2012). Categorical variables were analyzed with frequency and percentage; means with standard deviation were calculated for age. Qualitative type of analyses was done with statistical Chi-Square test P-Value < 0.05 was considered significant.

## RESULTS

Out of the total patients included in the study, pregnant women were 59(30.10%), their gestational period varied between 28-40 weeks, while delivered women were 137(69.89%), their postnatal period varied between day one to 42 days. Majority of these women were between 21-30 years of age 80(40.81%). Mean age±SD was 26.52±5.993. The most affected group was grand multiparous women, para four and above 83(42.34%) (P-Value<0.001). Pregnant women presented commonly during 33-37 weeks gestational period 32(54.23%) (P-Value 0.611), while delivered women were frequently presented within 1^st^ 24 hours of delivery 91(66.42%) (P-Value 0.235).(Table-I).

**Table-I:** Sociodemographic characteristics (N=196).

*S/NO*	*Sociodemographic characteristics*	*No of cases*	*Percentage*	*P-Value*
1.	***Age***			
	Mean age±SD: 26.52±5.993		
	a.upto 20 years	48	24.48
	b.21-30 years	80	40.81
	c.31 years and above	68	34.69
2.	***Parity***			
	a.primi	35	17.85	<0.0001
	b.para 1-3	78	39.79	
	c.para 4 and above	83	42.34	
3.	***Gestational period(pregnant women=59)***			
	a.28 -32 weeks	11	18.64	0.611
	b.33-37 weeks	32	54.23	
	c.38 weeks and above	16	27.11	
4.	***Post natal period (Delivered women=137)***			
	a. With in 1st 24 hours	91	66.42	0.235
	b.With in week	35	25.54	
	c.with in 42 days	11	8.02	

**Fig.1:**
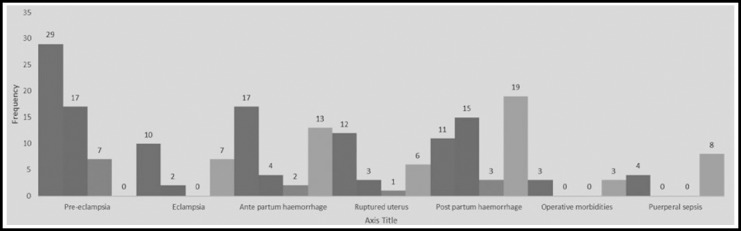
Management out come.

On general examination anaemia was found in 139(70.91%)(P-Value 0.163) while generalized oedema was seen in 123 (62.75%)(P-Value 0.174). The common clinical presentation was in the form of oligouria 92 (46.93%)(P-Value 0.061), anuria 68(34.69%), status of shock 59(30.10%) (P-Value 0.194), and fits 38(19.38%). Investigation report showed highest blood urea level more than 151mg/dl in 78 (39.79%) women(P-Value 0.165), serum creatinine level above 7mg/dl in 63(32.14%) women(P-Value 0.394), creatinine clearance level above 251ml/min in 39 (19.89%) women, serum uric acid level 12mg/dl in 40 (20.40%) women. Ultrasound examination report showed severe degree of renal parenchymal changes in 45(22.95%) women (P-Value 0.663).(Table-II).

**Table-II:** Clinical spectrum, Investigations (N=196).

*S/NO*	*Clinical Spectrum, Investigations*	*No of cases*	*Percentage*	*P-Value*
1	a.Anaemia	139	70.91	0.163
	b.Anuria	68	34.69	0.061
	c.Generalized oedema	123	62.75	0.174
	d.Oliguria	92	46.93	0.061
	e.Conciouness status:			
	i. Shock	59	30.10	
	ii.Fits	38	19.38	0.194
	iii.Restless, Irritable	22	11.22	
	f.Dyspnoea	17	8.67	
	g.Pulmonary oedema	13	6.63	0.966
	h.Increased bleeding tendencies	12	6.12	
2	***Investigations***			
	***a. Blood urea level***			
	i.50-100 mg/dl	51	26.02	0.165
	ii.101-150 mg/dl	67	34.18	
	iii.151mg and above	78	39.79	
	***b.Serum creatinine level***			
	i.2-3 mg/dl	55	28.06	0.394
	ii.4-6 mg/dl	78	39.79	
	iii.7mg and above	63	32.14	
	***c.Creatinine clearance level***			
	i.150-200 ml/min	18	9.18	0.335
	ii.201-250 ml/min	57	29.08	
	iii.251 ml/min and above	39	19.89	
	***d.Serum uric acid level***			
	i.6-8mg/dl	63	32.14	0.009
	ii.9-11 mg/dl	53	27.04	
	iii.12mg/dl and above	40	20.40	
	iv.Normal	40	20.40	
	***e.Ultrasound findings***			
	i.Normal renal parenchyma	49	25	0.663
	ii.Mild renal Parenchymal changes	43	21.93
	iii.Moderate renal parenchymal changes	59	30.10
	iv.Severe renal Parenchymal changes	45	22.95

Most frequent obstetrical emergencies were Pre eclampsia 53(27.04%), Post partum Haemorrhage 48(24.48%), Ante partum haemorrhage 36(18.36%), Ruptured uterus 22(11.22%), Eclampsia 19(9.69%), Puerperal sepsis 12(6.12%), andoperative morbidities were 6(3.06%). Complete recovery was observed in women commonly with eclampsia 29(14.79%), ante partum haemorrhage 17(8.67%), ruptured uterus 12(6.12%), post partum haemorrhage 11(5.61%), eclampsia 10(5.10%). Mortality rate was highest with post partum haemorrhage 19(9.69%), ante partum haemorrhage 13(6.63%), puerperal sepsis 8(4.08%), eclampsia 7(3.57%), ruptured uterus 6(3.06%).(Table-III).

**Table-III:** Acute renal morbidities with obstetrical emergencies and management outcomes (N=196).

*S/No*	*Obstetrical emergencies with renal morbidities and mortalities*	*Management outcomes*			

		*Completely recovered*	*Partially recovered*	*Lost follow up*	*Mortality*
1	Pre eclampsia 53(27.04%)	29(14.79%)	17(8.67%)	7(3.57%)	-
2	Eclampsia 19(9.69%)	10(5.10%)	2(1.02%)	-	7(3.57%)
3	Ante partum haemorrhage 36(18.36%)	17(8.67%)	4(2.04%)	2(1.02%)	13(6.63%)
4	Ruptured uterus 22(11.22%)	12(6.12%)	3(1.53%)	1(0.51%)	6(3.06%)
5	Post partum Haemorrhage 48(24.48%)	11(5.61%)	15(7.65%)	3(1.53%)	19(9.69%)
6	Operative morbidities 6(3.06%)	3(1.53%)	-	-	3(1.53%)
7	Puerperal sepsis 12(6.12%)	4(2.04%)	-	-	8(4.08%)

Total Grand	196	86(43.87%)	41(20.91%)	13(6.63%)	56(28.57%)

## DISCUSSION

Obstetrical acute renal failure is a rare entity in developed world. In the early days of dialysis, obstetric renal failure was a major part of the work of a renal unit. Acute renal failure was estimated to occur in one in 1400 to one in 5000 pregnancies in the UK; over the next 20-30 years medical complications of pregnancy remained a regular and frightening reason for acute renal referrals. Hammersmith Hospital in West London had a special interest in acute renal failure from the 1940s, and attracted many referrals. In their 1965 report by Smith K et al study^10^ obstetric renal failures accounted for 15% of cases requiring dialysis between 1957 and 1965, but it is still higher in developing countries like Pakistan. This is commonly observed in pregnant women as well as in the women in their puerperal period.

In this study acute renal morbidities with obstetrical emergencies were observed mostly in younger women having mean age±SD:26.52±5.993, grandmultiparous women (Para 4 and above)83(42.34%), mostly these referred women were delivered 137(69.89%), while pregnant women were 59(30.10%), these women were in their late pregnancy and with obstetrical emergencies. This is inconsistent with Nigerian study.^11^ This difference could be due to the quality of referral cases as this hospital is main tertiary care referral hospital of whole interior province receiving patient in emergencies. There is strong association between occurrence of any delay and poor maternal outcomes with life threatening conditions^12^, where as Joseph KSet al. study^13^ reported that pregnant women with acute renal failure at appropriate gestational period, prompt delivery will prevent further detoriation of the condition.

The general condition of these women was poor, majority of these women presented with oedema 123(62.75%), oliguria92(46.93%), anuria68(34.69%), status of shock 59(30.10%). On investigation highly raised blood urea level above 151mg/dl was reported in 78(39.79%), serum creatinine level above 7mg/dl in 63(32.14%). Ultrasound examination report showed severe degree renal parenchymal changes in 45(22.95%) women, same is reported by Piccoli GB et al study.^14^

In this study complete recovery rate was 86 (43.87%), this is inconsistent with Gurrier C et al study^15^ where it is reported thatthe majority of pregnancy-associated kidney injuries were transient; this difference could be due to the early diagnosis, proper referral, and appropriate management. Low complete recovery rate in this study could be due to the late referral, unbooked status, poverty, poor general health, and mismanagement at local maternity units same could be the reason of high mortality rate in this study 56(28.57%) with associated obstetrical emergency conditions like postpartum haemorrhage, ante partum haemorrhage, puerperal sepsis, eclampsia, ruptured uterus, these were the women who were anaemic, late referral, and in morbid condition, same is reported by other studies.^16^-^20^ The bias in study can be the no screening tests about renal function during pre pregnancy period as well as during pregnancy tenure for assessing renal status so that in high risk cases for subsequent obstetrical emergencies appropriate and immediate intervention with multidisciplinary approach could decrease morbidity and mortality.

## CONCLUSION

Renal morbidity and related consequences are the major events with obstetrical emergencies. Most common obstetrical emergencies leading to renal morbidities and associated mortalities were pregnancy induced hypertensive disorders, antepartum and postpartum haemorrhage, ruptured uterus, puerperal sepsis. Appropriate preventive measures such as early identification of at risk women, proper referral for providing multidisciplinary services at the tertiary level, optimization of fluid balance, identification and treatment of cause, delivery at appropriate gestational period, and timely initiation of renal replacement therapy may reduce mortality.

### Authors’ Contribution

***Meharunnissa Khaskheli:*** Concept, designed the study and is accountable for all the work.

***Shahla Baloch:*** Contributed in drafting the manuscript.

***Aneela Sheeba:*** Critically revised for important intellectual content.

***Sarmad Baloch:*** Helped in data analyses.

***Fahd Khan:*** Helped in acquisition and interpretation of data.

***M. Rafique Ansari:*** Approval of the final version to be published.
